# Induction of Human Squamous Cell-Type Carcinomas by Arsenic

**DOI:** 10.1155/2011/454157

**Published:** 2011-12-06

**Authors:** Victor D. Martinez, Daiana D. Becker-Santos, Emily A. Vucic, Stephen Lam, Wan L. Lam

**Affiliations:** Department of Integrative Oncology, BC Cancer Research Centre, 675 West 10th Avenue, Vancouver, BC, Canada V5Z 1L3

## Abstract

Arsenic is a potent human carcinogen. Around one hundred million people worldwide have potentially been exposed to this metalloid at concentrations considered unsafe. Exposure occurs generally through drinking water from natural geological sources, making it difficult to control this contamination. Arsenic biotransformation is suspected to have a role in arsenic-related health effects ranging from acute toxicities to development of malignancies associated with chronic exposure. It has been demonstrated that arsenic exhibits preference for induction of squamous cell carcinomas in the human, especially skin and lung cancer. Interestingly, keratins emerge as a relevant factor in this arsenic-related squamous cell-type preference. Additionally, both genomic and epigenomic alterations have been associated with arsenic-driven neoplastic process. Some of these aberrations, as well as changes in other factors such as keratins, could explain the association between arsenic and squamous cell carcinomas in humans.

## 1. Background

Arsenic is a well-known human carcinogen (Class I, according to the International Agency for Research on Cancer—IARC [1]). Recommended thresholds for arsenic concentration in drinking water is ≤10 *μ*g/L [2, 3] however, chronic exposure exceeding this limit has been reported worldwide affecting nearly one hundred million people worldwide daily, especially in Bangladesh, Taiwan, Mongolia, India, China, Argentina, Mexico, Canada, USA, and Chile, among others countries [4–9]. Long-term effects are a major health concern in affected areas. 

In the environment, arsenic can be found with an oxidation state +3 (known as arsenite or As [III]), or +5 (arsenate or As [V]), exhibiting different grades of toxicity [10]. Increased levels of inorganic arsenic (combined with oxygen, chlorine, and sulfur, among other elements) in drinking water is the major cause of arsenic toxicity [11, 12].

Both neoplastic and nonneoplastic effects have been described as consequence of arsenic exposure. Nonneoplastic effects primarily include peripheral vascular disorders (e.g., “black foot disease” almost exclusively observed in zones of Taiwan affected by arsenic contamination [13]), hypertension, diabetes, severe atherosclerosis, neuropathies, and, importantly, skin alterations, such as hyperkeratosis and hyperpigmentation [13–16]. Hyperkeratosis is particularly relevant, because it has been described as a precursor lesion of skin cancer tumors associated with arsenic exposure [17–19].

Several types of cancer have been attributed to arsenic exposure [20–25]. Skin, bladder, and lungs are the major target organs affected by different routes of exposure. Skin cancer is the most common form of malignant tumor related to arsenic ingestion. Lung cancer is the most deadly form of cancer associated to arsenic exposure [26, 27]. For internal organs, bladder cancer exhibits the highest relative risk [20, 28, 29]. Other less common types of cancer associated with arsenic exposure include kidney, liver, and prostate cancers [30, 31].

The mechanism of arsenic-induced diseases remains to be elucidated. When arsenic enters the human body, it is readily reduced from the pentavalent arsenical species to trivalent forms, and, subsequently, there are a series of oxidative methylation reactions leading to the generation of tri- and pentavalent methylated metabolites [32]. It has been proposed that the biotransformation processes of arsenic activate its carcinogenic potential. This hypothesis was supported by the observation that the methylated arsenical species (specially trivalents) exhibit aberrant effects such as enzyme inhibition (particularly oxidoreductases), damage to DNA structure, and activation AP-1-dependent gene transcription [33, 34].

Neoplastic effects as a result of arsenic biotransformation can occur at the genetic and epigenetic levels. Arsenic can damage DNA, mostly through oxidative stress by generation of toxic species such as reactive oxygen species (ROS), which can potentially lead to genomic aberrations.

**Figure 1:**
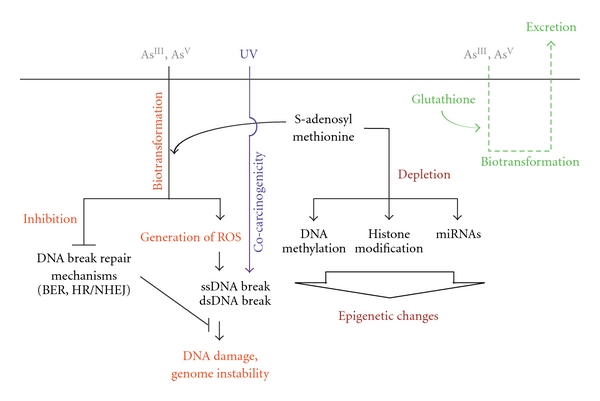
Carcinogenic mechanisms of arsenic transformation. Ingested arsenic undergoes a biotransformation process. (1) Biotransformation could lead to arsenic excretion, when conjugated with glutathione. (2) Biotransformation generates reactive oxygen species (ROS), namely, superoxide anions (O_2_^−^), hydrogen peroxide (H_2_O_2_), hydroxyl radicals (OH), that induce single-strand (ssDNA) and double-strand (dsDNA) breaks by inducing oxidative damage. The process can also inhibit DNA break repair mechanisms for ssDNA breaks (base excision repair (BER)) and for dsDNA breaks (homologous recombination (HR) and/or nonhomologous end joining (NHEJ)). Additionally, ROS derived from arsenic biotransformation can act as cocarcinogens, for example, increasing damage potential of ultraviolet (UV) light. Furthermore, the requirement of S-adenosyl methionine (SAM) for arsenic biotransformation can lead to depletion of SAM, which is the substrate for DNA methylation.

Figure 1 Secondly, arsenic uses S-adenosylmethionine (SAM) as a methyl group donor during its biotransformation. As a consequence, deprivation of the cellular pool of methyl groups can block certain cellular processes, resulting in alteration of epigenetic mechanisms that can contribute to arsenic-induced carcinogenesis [35]. Arsenic and arsenic metabolites can directly and indirectly affect normal epigenetic transcriptional regulation at the level of DNA methylation, histone maintenance, and miRNA expression (Figure 1) [36, 37].

### 1.1. Cell-Type Specificity on Arsenic-Induced Cancers

Despite physiological differences among target organs for arsenic carcinogenicity, a common characteristic has been described, namely, a squamous cell-type preference, independent of the affected organ. In fact, SqCC is one of the most common cancers seen in chronic-arsenic-exposed humans [38, 39]. Cell-type specificity has been previously described mainly for skin and lung cancer, and some studies have shown associations with liver cancer [40, 41]. In this paper, we will focus on specificity reported in two main arsenic-associated types of cancer, namely, skin and lung cancers.

### 1.2. Skin Cancer and Arsenic Exposure

Skin cancer is the most common malignancy associated with arsenic ingestion through drinking water [26]. There are three major pathological cell types of skin cancer: basal cell carcinoma (BCC), squamous cell carcinoma (SqCC), and malignant melanoma. The first two cell types are the most common types of skin cancer, and they belong to the group of nonmelanoma skin cancers. Exposure to arsenic in drinking water has been associated with the incidence of SqCC and BCC, but not with the incidence of malignant melanoma [42]. Arsenic-related SqCC of the skin can develop either *de novo* or progress from Bowen's disease (BD), a *premalignant skin disorder*, frequently reported as a consequence of chronic arsenic exposure [43–46]. Additionally, there are differences between skin cancer cases induced and not induced by arsenic. For example, arsenic-related BCC develops typically in non-sun-exposed areas of the body (contrary to UV-related skin cancer) and exhibits multiple foci [47].

Cell type specificity has been directly observed in chronically exposed human populations. In a key study from Taiwan, SqCC and BCC appear to be associated with ingestion of arsenic [42]. However, prior to skin cancer, the first observed effects of chronic arsenic exposure were nonmalignant skin lesions, such as hyperpigmentation and hyperkeratosis [43, 48]. Skin hyperkeratosis is common in chronic arsenicosis and is considered a precursor of SqCC because cancer can arise from these lesions [17–19].

Arsenic tends to concentrate in ectodermal tissue including the skin, hair, and nails [48]. Dermal absorption of arsenic is not the main exposure route, since some derivates of arsenic metabolism have low permeability [49, 50]; however, both arsenite and arsenate accumulate in the dermis and epidermis [49]. In this context, epidermal stem cells have been proposed as a potential target for arsenic-induced carcinogenesis, since this metalloid can increase the relative proportion of stem cells in culture [51, 52].

Keratinocyte stem cells (KSCs) are a group of relatively quiescent cells with a broad proliferative potential and an unlimited capacity for self-renewal [53, 54].* DSS1* (*deleted in split hand/split foot 1*) is a 12-O-tetradecanoyl phorbol-13-acetate-(TPA-) inducible gene expressed in KSCs that plays an important role in skin carcinogenesis and is required for epidermal cell proliferation and oncogenic transformation [55]. *DSS1 *expression was significantly increased in SqCC induced in adult skin of Tg.AC mice—a strain sensitive to skin carcinogenesis via activation of the v-Ha-ras transgene likely in KSCs—by arsenic plus TPA compared with TPA alone [56]. Other genes responding to the same activation mechanisms in skin cancer, such *Sprr2a*, *Ptges*, and *Col1a2,* were also increased in SqCC developed in Tg.AC mice [56, 57]. Arsenic also has the potential to induce malignant transformation of human keratinocytes. Immortalized human epithelial cell line (HaCaT) exposed to 100 nM of arsenic over 28 weeks generated highly aggressive SqCC when inoculated into nude mice [58].

Matrix metalloproteinase 9 (MMP-9) plays an important role in tumor progression, specifically in metastatic capacity, and its constitutive secretion correlates with the degree of tumorigenicity in human keratinocytes [59, 60]. Human keratinocytes transformed by arsenic exhibit an increased secretion of MMP-9, to a similar degree observed in SqCC generated after inoculation of arsenic-transformed cells in mice [58]. This indicates that an aberrant expression of MMP-9 could be an early marker for arsenic-induced skin SqCC.

Finally, it has been suggested that arsenic can act as a skin cocarcinogen (Figure 1). In this context, it has been demonstrated that low concentrations of arsenic can increase tumor induction by UV-light in mice, with a high proportion of SqCC [61].

### 1.3. Lung Cancer and Arsenic Exposure

Lung cancer is the most deadly form of cancer associated to arsenic ingestion [27]. Several reports have shown a higher incidence of lung SqCC in populations exposed to arsenic (Table 1). This relationship has been demonstrated in several human populations. In Taiwan, patients from an endemic area of arsenic intoxication with exposures through drinking water had higher proportions of squamous cell and small cell carcinomas, but a lower proportion of adenocarcinomas [40].

In northern Chile, a high proportion of SqCC frequently occurs in never smokers who have been chronically exposed to arsenic, which is interesting, since SqCC is very frequently associated with cigarette smoking and its incidence has decreased worldwide [24, 62]. In rural areas of Bangladesh, arsenic contamination in drinking water from tube wells is associated with lung cancer in males, with lung SqCC being the predominant histological subtype in areas with arsenic concentrations above 100 *μ*g/L [63]. Additionally, lung SqCC was much more frequent among Bangladesh inhabitants who smoke and are exposed to arsenic in drinking water (concentrations >100 mg/L) compared to nonsmokers [63].

Studies in people occupationally exposed to arsenic have also revealed cell type specificity for arsenic-induced lung cancer. A *postmortem* study found that 37.6% of lung cancers among wine growers affected by chronic arsenic poisoning were of the SqCC type, a proportion significantly higher compared with three of the four reference populations [64]. On the other hand, smelter workers exposed to arsenic did not exhibit major changes in histological distribution for lung cancer (compared with reference group); however, lung SqCC has the highest number of cases, even among never smokers [65, 66]. Conversely, other studies have shown no differences in histological types of lung cancer associated with arsenic exposure, challenging the hypothesis that small cell undifferentiated and epidermoid (SqCC) carcinomas are the only subtypes that increase in response to arsenic, among other carcinogens [67].

Information from German uranium miners who died from lung cancer during 1957–1990 was analyzed under the hypothesis that arsenic may influence the distribution of lung cancer types [68]. There was an arsenic-related increase in the proportion of lung SqCC among miners without silicosis at all levels of coexposure to radon and quartz dust.

### 1.4. Role of Keratins in Arsenic-Induced SqCC

The main structural proteins of the intermediate filament-based cytoskeleton of epithelial cells are the keratins [69]. Keratins are closely related with tissue-specific and cell-specific differentiation of epithelial cells [70], accordingly their roles in arsenic-induced SqCC have been explored. Interestingly, arsenic-induced carcinogenesis is related to enhanced expression of these cytoskeletal proteins in skin, liver, and bladder [41, 58, 71–73].

Hyperkeratinization of stratified epithelia is a common clinical manifestation of arsenic exposure [17]. Notably, arsenic-associated SqCC typically arises from arsenic-induced hyperkeratotic lesions, suggesting that dysfunctional keratinization may play a critical role in arsenic-induced carcinogenesis [18]. In human keratinocytes, arsenic has been shown to promote a dramatic increase in expression of CK1, CK10, involucrin, and loricrin [58], which are major markers of squamous differentiation [74]. The epidermal stem cell markers CK5, CK14, and CK15 have also been shown to increase with arsenic exposure [72, 75–77]. CK8, CK17, and CK18 which are overexpressed in invasive SqCC, are also induced in arsenic-treated keratinocytes [72]. In addition to cell model studies, a population-based report of chronic arsenic exposure in Taiwan revealed progressive alterations in keratin expression in various skin lesions, including hyperkeratosis and SqCC. In this study, CK6, CK16, and CK17 were associated with arsenic exposure in SqCC [73].

The disruption in keratins induced by arsenic promotes the formation of DNA-protein cross-links, which can impair DNA replication [41]. The fact that arsenic has a high affinity to thiol groups, including those attached to thiol-rich proteins such as keratins, might contribute to its accumulation in epithelial tissues leading to further DNA-protein cross-links, DNA damage, and possibly other defects. Indeed, keratin synthesis is tightly correlated with differentiation programs of a range of epithelial cell types [78]. Therefore, arsenic could not only damage DNA but also modify differentiation patterns in the cells where it accumulates. The subsequent arsenic-induced genetic and epigenetic alterations could finally lead to the development of SqCC.

### 1.5. Genetic and Epigenetic Alterations Associated with Arsenic Exposure

Some genetic alterations have been described to be specifically associated to arsenic ingestion in humans. For example, Moore et al. identified genomic changes in bladder tumors (transitional cell carcinoma, TCC) from arsenic-exposed patients using chromosomal comparative genomic hybridization (CGH) [79]. Among different recurrent arsenic-related DNA copy number alterations (CNAs) identified, DNA loss of chromosome 9q is remarkable, since it was the only alteration more frequently observed in never smokers than in ever smokers in this study. Similarly, Hsu et al. identified CNAs on bladder TCC tumors from southwest Taiwan [80]. Gain at 3p and loss at 17p13 were consistent with findings from northern Chile and Argentina.

**Figure 2:**
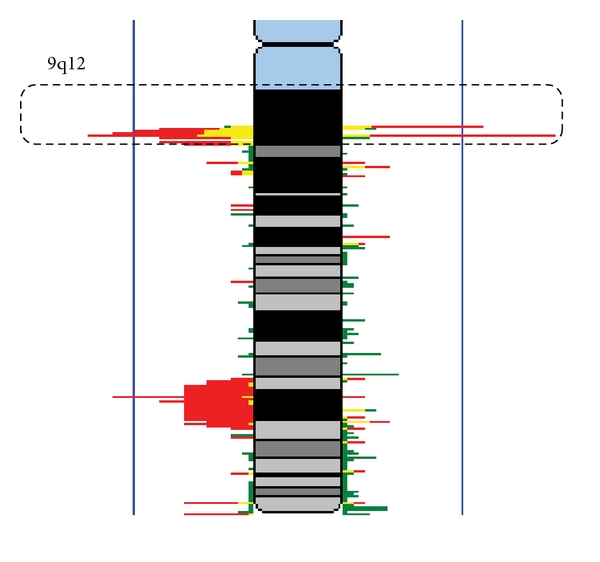
DNA copy-number alteration at chromosome 9q in arsenic-exposed lung SqCC. CNA frequencies are overlaid in this karyogram. Profiles of 52 lung SqCC biopsies were generated using a submegabase resolution tiling-set rearray (SMRTr) platform. Twenty-two arsenic-exposed samples (shown in red) were obtained from lung cancer patients from northern Chile. Thirty samples from patients without known arsenic exposure (green) were obtained from North American individuals. Regions in yellow denote a sector of overlapping alteration status in both groups. The magnitude of red, green, and yellow bars represents percentage of samples (0–100%, with blue vertical lines representing 50% frequency) exhibiting DNA gains (to the right) and DNA losses (to the left). Dotted line represents 9q12 cytoband.

Recently, arsenic-related CNAs were identified among lung SqCCs from chronically exposed patients from northern Chile [81]. Alterations were identified by a whole genome tiling-path array comparative genomic hybridization (CGH) platform (described in [82, 83]). Noticeably, a 0.3 Mb DNA loss at 9q12 was preferentially detected among arsenic-exposed cases (Figure 2). The role of chromosome 9q on arsenic-induced human tumors needs to be further investigated; however, common alterations detected among bladder and lung tumors indicate a potential participation on arsenic-related neoplastic process.

Arsenic also has epigenetic effects at the level of DNA methylation, histone maintenance, and miRNA expression (reviewed in [36, 37, 84]). Since arsenic biotransformation uses S-adenosylmethionine (SAM) as a methyl group donor, it has been proposed that SAM depletion can interfere with cellular processes that require methyl groups, leading to the idea that alteration of epigenetic mechanisms can also participate in arsenic-induced carcinogenesis [35]. Promoter hypermethylation affecting *P53, p16INK4A, RASSF1A,* and *PRSS3* has been described in lung cancer cell lines and tumor tissues from mice and human [85–87]. On the other hand, arsenic can modify normal histone patterns. As [III] has also been shown to increase H3K9 dimethylation and decrease H3K27 trimethylation, marks associated with heterochromatin formation (i.e., gene silencing) and a decrease in H3K4 trimethylation which is associated with actively transcribed euchromatin [88]. In human nontumorogenic cell lines, arsenic-induced malignant transformation provoked changes in expression for specific genes, which was correlated with the histone acetylation levels for their respective promoter regions [89].

In the few past years, miRNAs have been intensively studied in the context of arsenic-related cancer. An increasing number of studies show that arsenic exposure can alter miRNA expression levels *in vitro* and *in vivo*. miRNA alterations associated with arsenic (or its metabolites) have been demonstrated in human lymphoblastoid cells, human peripheral blood-derived cells, and chick embryos [87, 90]. Exposure of immortalized human bronchial epithelial cells to low levels of arsenic induced malignant transformation and epithelial-to-mesenchymal transition in p53- dependent manner by reducing levels of miR-200 family members [91]. In the case of human peripheral blood-derived cells, changes in miRNA could be related with changes in methylation patterns, since the same alterations were observed when cells were grown under folate-deficient conditions, which can lead to reduced levels of SAM [87].

## 2. Conclusion

Arsenic in drinking water affects millions of people worldwide, posing an urgent health concern [92]. Hundreds of millions of people have been exposed to this metalloid through drinking water, resulting in morbidity and mortality globally [5, 6]. Arsenic causes a variety of health problems, significantly cancer, whose symptoms may not appear for 20 to 30 years after exposure [93].

Several genomic aberrations have been described for arsenic-induced cancers. Based on distinctive profiles of genomic changes, it has been proposed that arsenic-induced lung SqCC could correspond to a molecularly distinct form of lung cancer [81]. Additionally, the arsenic biotransformation process is associated with a series of methylation changes, which has led to the hypothesis that epigenetic changes play a key role in arsenic-induced carcinogenesis. Changes in DNA methylation patterns (especially at promoter regions of key genes) and alterations to the “histone code” are likely mechanisms of arsenic-induced malignant transformation. Additionally, miRNAs seem to play an important role at this level.

Understanding the mechanisms underlying the preferential occurrence of squamous cell-type tumors associated with arsenic exposure, especially skin and lung cancer, is an urgent health care issue. Arsenic has been shown to promote a dramatic increase in expression of keratins related to squamous differentiation, which can be, in part, a consequence of genetic and epigenetic changes associated with arsenic biotransformation. This corresponds to a potential mechanism for explaining the preferential histological subtype induced by arsenic, independent of the target organ. Further research is needed in order to clarify the molecular mechanisms governing arsenic-driven carcinogenesis, as well as preference for histological subtypes. This knowledge can improve strategies oriented to early diagnosis or targeted therapies of these and other arsenic-related diseases.

## Figures and Tables

**Table 1:** Studies exhibiting associations between arsenic exposition and cell types of lung cancer.

Location	Number of lung cancer cases	Cell type	Smoking status	Calculated risk	Dosage	Reference
Britain	6 cases treated with Fowler's solution or potassium arsenite (2 male/4 female)	(i) 5 undifferentiated carcinomas (ii) 1 SqCC	3 never smokers, 1 smoker	NA	NA	[94]

Taiwan	(i) 76 LC cases (50 males/36 females) (ii) 400 controls from the same administrative area	(i) Male cases: 40% SqCC, 38% AdC, 22% others (ii) Female cases: 19% SqCC, 69% AdC, 11% others	NA	Odds ratios for developing LC were 3.39 for whom used well water from arsenic-contaminated zones during 40 or more years, compared with people who never used such water source	Median of 780 *μ*g/L (artesian wells) and 40 *μ*g/L (in well water)	[95]

Japan: Niigata Prefecture	(i) 443 individuals exposed to arsenic (ii) 9 (8 male/1female) developed lung cancer after exposure to high levels (≥1000 *μ*g/L)	(i) 3 SCC (ii) 2 SqCC (iii) 1 SqCC + SCC	All smokers, except for female case	SMR = 15.6 for developing LC among individuals (*N* = 113) exposed to >1000 *μ*g/L of arsenic in drinking water in a zone contaminated during 5 years in Japan, while individuals exposed to 50−990 *μ*g/L (*N* = 76) present a SMR of 2.33		[96]

Southwest and northeast Taiwan	139 newly diagnosed LC cases from a BFD endemic zone	(i) 45% SqCC (ii) 22% AdC	(i) 31.9% never smokers (ii) 16.7% past smokers (iii) 51.4% current smokers	Relative risk according to exposure group (i) Group 1: 1 (ii) Group 2: 1.09 (iii) Group 3: 2.28 (iv) Group 4: 3.03 (v) Group 5: 3.29	Average arsenic level in groundwater (*μ*g/L) (i) Group 1: <10 (ii) Group 2: 10–99l (iii) Group 3: 100–299l (iv) Group 4: 300–699 (v) Group 5: ≥700	[97]

Taiwan	National Cancer Registration Program with 37.290 LC patients (26.850 men/10.440 women	Men^†^ (i) 33.6% SqCC (ii) 19.5% AdC Women^†^ (i) 29.9% SqCC (ii) 29.6% SqCC	NA	Male and female patients from the BFD area had higher proportions of SqCC (RR = 1.1 in men and RR = 1.9 in women) and SCC (RR = 1.2, in men, and RR = 2.3, in women) but had a lower proportion of AdC (RR = 0.7 in men and RR = 0.6 in women)	(i) Towns in BFD area: average arsenic level of 0.22 mg/L in well water (ii) Control towns: average arsenic level of 0.02 mg/L	[40]

Bangladesh	(i) 3.223 (2811 male) with a primary LC (ii) 1588 (1183 male) with benign lesions	50.5% and 39.0% of SqCC among smokers and nonsmokers, respectively	(i) 79.7% smokers (ii) 18.5% never (iii) 1.80% unknown	OR = 1.45, (95% CI 1.16−1.80)	>100 *μ*g/L	[63]

Northeastern Taiwan	(i) 8086 residents were followed for 11 years (6888 remained in the final analysis) (ii) 178 incident LC cases	(i) 75 (42.1%) SqCC (ii) 51 (28.7%) AdC (iii) 22 (12.4%) SCC	At enrollment (i) 59.0% never smokers (ii) 12.4% past (iii) 28.6% current	The RRs and 95% CIs for 100–300 and >300 *μ*g arsenic/L when compared with <10 *μ*g arsenic/L were 1.54 (0.97–2.46) and 2.25 (1.43–3.55), respectively	The mean (arsenic) among wells with known arsenic concentration was 117.2 *μ*g/L	[98]

USA: New Hampshire, Vermont.	(i) 223 lung cancer (100 male/123 female) (ii) 238 controls	75 cases were SqCC and SCC	Data from cases (i) 5.4% never smokers (ii) 94.6% current	Arsenic exposure was associated with SCC and SqCC (OR = 2.75 for toenail arsenic concentration ≥0.114 *μ*g/g versus <0.05 *μ*g/g)	Toe nail (arsenic) in 4 levels: <0.05 *μ*g/g, 0.05 to <0.0768 *μ*g/g, 0.768 to <0.1137 *μ*g/g, and ≥0.1137 *μ*g/g	[99]

^†^Data from patients from the Blackfoot Disease area in Taiwan.

SCC: small cell carcinoma; NA: data not available; SMR: standardized mortality rate; OR: odds ratios.
